# Exploring the impact of rural health system factors on physician burnout: a mixed-methods study in Northern Canada

**DOI:** 10.1186/s12913-021-06899-y

**Published:** 2021-08-25

**Authors:** Nathaniel Hansen, Kennedy Jensen, Ian MacNiven, Nathaniel Pollock, Thomsen D’Hont, Susan Chatwood

**Affiliations:** 1 Tufts University School of Medicine, Boston, MA USA; 2 Geisel School of Medicine at Dartmouth, Hanover, NH USA; 3 Northwest Territories Health and Social Services Authority, Yellowknife, Northwest Territories Canada; 4 School of Public Health, University of Alberta, Edmonton, Alberta Canada; 5 School of Arctic and Subarctic Studies, Labrador Institute, Memorial University, Happy Valley-Goose Bay, Newfoundland and Labrador Canada; 6 Department of Family Medicine, Faculty of Medicine, University of Alberta, Yellowknife, Northwest Territories Canada; 7 School of Public Health, University of Alberta, Yellowknife, Northwest Territories Canada

**Keywords:** Rural healthcare, Indigenous health, Physician burnout

## Abstract

**Background:**

Burnout among physicians is a consequence of chronic occupational stresses and emotionally intense work demands. However, much of the evidence exploring burnout is derived from urban settings and may not reflect the work and social contexts of physicians in Indigenous communities or in rural and resource-constrained areas. We sought to characterize health system factors that influence burnout among physicians practicing in the three northern territories of Canada.

**Methods:**

We conducted a mixed-methods study that included an online survey and qualitative interviews with physicians practicing in Nunavut, Northwest Territories, or Yukon in 2019. The survey adapted content from the Maslach Burnout Inventory. Results were analyzed with logistic regression to assess the association between health system factors and burnout. We conducted in-depth interviews with 14 physicians. Qualitative data was coded and analyzed for themes using the ATLAS.ti software.

**Results:**

Thirty-nine percent of survey respondents (*n* = 22/57) showed features associated with burnout. Factors associated with burnout included use of electronic medical records (β = − 0.7, *p* < .05), inadequate financial remuneration (β = − 1.0, *p* < .05), and cross-cultural issues (β = − 1.1, *p* < .05). Qualitative analysis further identified physician perceptions of lack of influence over health system policies, systemic failures in cultural safety, discontinuity of care, administrative burden, and physician turnover as important drivers of burnout.

**Conclusions:**

Physicians practicing in northern regions in Canada experience stress and burnout related to health system factors and cross-cultural issues. The relationship between cross-cultural issues and burnout has not previously been reported. This work may have implications for physician wellbeing and workforce attrition in other resource-constrained or culturally diverse clinical settings.

**Supplementary Information:**

The online version contains supplementary material available at 10.1186/s12913-021-06899-y.

## Background

Burnout among physicians is a consequence of chronic occupational stresses in medicine coupled with emotionally intense work demands [1, 2]. Adverse health outcomes associated with physician burnout include substance use, insomnia, and depression [3–5]. Burnout can lead to poor patient outcomes, in some cases doubling the risk of medical error and increasing recovery times for hospitalized patients [6, 7]. Burnout also has broader consequences for health systems as a contributor to reduced productivity and staff turnover [8]. A previous study from Canada estimated that early retirement and reduced clinical hours related to burnout increased health system costs due to a loss of services, especially among family physicians [9].

Much of the literature describing physician burnout has compared experiences across specialty, career stage, age, and gender. Nationally-representative surveys of physicians have allowed for comparisons across geographic contexts as well [10, 11], though comparatively limited work has described the specific experiences of physicians working in resource-constrained or rural settings [12–14]. A recent study of family physicians in the US found that there was no significant difference in rates of burnout between those practicing in rural and urban settings [11]. Similar analyses are possible with the Canadian National Physician Health Survey. However, physicians from the three northern territories of Nunavut, Northwest Territories (NWT), and Yukon, are often underrepresented in this survey [15]. These limitations are problematic because the context of healthcare in northern and rural Canadian provinces differs vastly from the clinical settings of southern cities where the majority of physicians practice [16].

### The social context of health in northern Canada

The geographic and demographic contexts in Northern Canada have important implications for health system accessibility and efficacy. Together, Nunavut, NWT, and Yukon cover an arctic and subarctic area of over 3 million kilometers square (Table 1) and comprise 39% of Canada’s total landmass. Outside of the territorial capitals, there are more than 80 communities in the territories, many of which are only accessible year-round by air, and seasonally by boat or snowmobile.

**Table 1:** Geographic and Demographic Characteristics of the Yukon, NWT, and Nunavut

Characteristics	Yukon	Northwest Territories	Nunavut
Land area (km^2^)	474,712	1,143,793	1,877,778
Number of municipalities	19	33	25
Population Territorial Capital	35,874 25,085 Whitehorse	41,786 19,569 Yellowknife	35,944 7740 Iqaluit
% Indigenous	19.1% First Nations 2.9% Métis 0.6% Inuit	32.1% First Nations 9.9% Inuit 8.2% Métis	84.7% Inuit 0.5% First Nation 0.5% Métis
Mean Age	39.1 years	34.9 years	27.7 years
Life Expectancy at Birth (2014–2016)	79.0 years	77.2 years	71.7 years

The population of the territories is relatively small (Table 1) and accounts for 0.3% of the national population. Inuit make up the majority of the population in Nunavut and nearly 10% in NWT; First Nations and Métis together account for 20 and 40% of the population in Yukon and NWT respectively. Indigenous languages are widely spoken in the territories – there are 8 Indigenous language groups in Yukon and 9 in the NWT; Inuktut is the official Indigenous language in Nunavut, and is spoken by 71% of the population.

Indigenous peoples in the territories experience substantial health inequities compared to Canada overall. During the 2014–2016 period, life expectancy in the territories (Table 1) was between 3 and 10 years lower than in Canada overall [17]. Similar gaps between northern Indigenous populations and the general population are evident for other key population health indicators including premature mortality due to injuries and suicide, hospitalization for ambulatory care sensitive conditions, and rates of diabetes, tuberculosis, and cancer [18–21]. These inequalities are proximally related to inequities in the social determinants of health, but have their upstream origins in structural determinants related to colonization [22, 23].

Health services in the North are delivered under a system of universal coverage by the territorial governments, though Indigenous governments and the federal government also provide community-based healthcare, mental health, and social services in some communities. Federal legislation in Canada requires that healthcare be not only universal, but also publicly funded and administered, accessible, comprehensive, and portable. Despite these goals, Indigenous populations, especially in the North, experience systemic barriers to healthcare access and quality including travel, long wait times, language, and provider turnover. Many Northern communities also experience difficulties with recruitment and retention of health professionals, often relying on short-term coverage by locum providers. Additionally, northern health systems are consistently constrained by underfunding, which exacerbates challenges of providing care across large geographic areas. Disparities in access are starkly evident in family medicine: while 85% of Canadians have access to a family doctor, only 74% of Yukon residents, 42% of NWT residents, and 17.5% of Nunavut residents do [24]. The resulting inequities represent the cumulative consequences of the current approach to healthcare delivery in the northern context.

### Objective

Recent qualitative studies have shown that physicians in northern and rural regions of Canada often face unique clinical challenges related to overcoming resource constraints and working outside of the scope of their practice to provide care that best serves both patients and communities [25, 26]. Given the broader context of health in the North, physicians may be exposed to context-specific risks for burnout that are not captured or well understood in the existing literature. The objective of this study was to examine the relationships between structural factors in the health system, including governance, organization, and work environment-related issues, and physician burnout in northern Canada.

## Methods

We conducted an explanatory sequential mixed-methods study that combined a cross-sectional survey and in-depth qualitative interviews [27]. We chose a mixed-methods approach because it allowed us to characterize burnout and its contributing and protective factors with a standardized measure while also exploring the perspectives and experiences of physicians in an effort to explain the context for burnout in northern regions. Populations in rural and remote contexts are often small, yet the findings of research conducted in these settings remain important and should not be hindered by an inability to generate large statistical power. Given the size of our target population and the existing work-related constraints on their time, we anticipated a sub-optimal response rate. In this context, a mixed-methods approach was an appropriate way to address our research question with greater accuracy and detail, as has been previously demonstrated in other rural settings [16]. This study was approved by the Aurora College Research Ethics Committee. We used GRAMMS guidelines to report the results [28].

### Setting

We conducted this study with physicians in the three northern territories in Canada – Nunavut, NWT, and Yukon. Physician services are publicly funded through a health transfer from the federal government to the territorial governments. Remuneration schedules in the territories vary by specialty and practice setting, and include both salary and fee-for-service models.

Each territory has a general hospital with specialty services including obstetrics, pediatrics, internal medicine, surgery, and psychiatry. Family physicians comprise the largest sector of the medical workforce in the territories, and have a broad scope of practice that includes primary care, low-risk obstetrics, hospitalist, emergency, palliative care, aeromedical evacuations, and surgical assist.

Physician services are concentrated in the territorial capitals, though some family physicians are based in regional health centers. For remote communities, nurses and nurse practitioners provide care in community clinics, and rely on telehealth and medical transportation to help patients access physician services. For most subspecialty care, patients from the territories must travel to tertiary care centers in southern cities. Most physicians in the territories are non-Indigenous, not from the North, do not speak an Indigenous language, and received their medical education and training outside of the territories.

### Target Population

The target population for this study was physicians from any specialty who were licensed, in practice, and residing in Nunavut, NWT, or Yukon as of September 2019. Locum physicians were not eligible for the study. The candidate population included 93 physicians in Yukon, 56 in the NWT, and 25 in Nunavut, as defined by the NWT Health and Social Services Authority and Yukon Medical Association (YMA) workforce lists.

### Data collection

The quantitative component utilized a two-step survey that integrated the Maslach Burnout Inventory (MBI) and a supplementary questionnaire. The MBI is a 22-item assessment that characterizes physician experiences on three subscales including emotional exhaustion, cynicism, and professional efficacy [29]. Scores on these subscales are then used to categorize participants into one of five distinct profiles: engaged, ineffective, overextended, disengaged, and burnout. Each profile is reflective of different workplace experiences and varying abilities to cope with workplace stressors. For example, an engaged profile would characterize a worker with minimal exhaustion and cynicism, and a strong sense of efficacy; this pattern describes a positive experience of work without signs of burnout [29]. All other profiles involve different degrees and manifestations of burnout.

The supplementary questionnaire collected information about demographics and responses to Likert questions about the perceived roles of health system factors in physician burnout. The burnout exposures in the supplementary questionnaire were drawn from existing literature with input from a co-author who is a staff physician in the NWT. The survey also contained open-ended questions that allowed participants to provide narrative responses.

For the qualitative phase of the study, our team developed a semi-structured interview guide (Additional file 1) that probed physicians’ reflections about burnout experienced, either personally or by colleagues, while practicing medicine in Northern Canada. We designed the guide to mirror the survey using broader open-ended questions. Interviews with individual participants were conducted by phone or video conference between September 2019 and April 2020 by one author (KJ), who was a medical student experienced in conducting qualitative research. The interviewer did not have prior relationships with participants, though had resided in the NWT prior to the study. All interviews were audio recorded with the consent of participants and transcribed verbatim by the interviewer. The interviewer maintained reflexive validity by recording her opinions and perceptions before beginning data collection. This bracketing process of documenting preconceptions at the outset minimized bias in interviews and analysis.

### Recruitment

To recruit participants to the survey, an invitation letter and link to the online survey was emailed to all eligible physicians (*n* = 174) by an author (IM), who lives in the NWT and is a practicing orthopedic surgeon. The letter explained that participation was voluntary and responses were anonymous. All participants provided informed consent at the beginning of the survey.

The overall response rate was 32% (*n* = 57), though this varied by territory: 12% in Nunavut, 77% in the NWT, and 11% in Yukon. The survey response in Nunavut was likely impacted by a territory-wide ransomware attack in November 2019 that restricted email and web access for territorial government employees and prevented some potential participants from accessing the survey. All survey participants were asked to indicate their willingness to participate in a follow-up interview.

Of the 57 physicians who completed the survey, 14 took part in the qualitative component of the study. Interviews were conducted with 3 physicians from Nunavut, 10 from the NWT, and 1 from Yukon. The interviews lasted between 25 and 40 min. Participants were not remunerated for taking part in any aspect of the study.

### Data analysis

For the survey data, we used descriptive statistics to summarize the demographic variables, then used the responses to the MBI questions to categorize participants based on the five distinct profiles from the engagement-burnout continuum [29]. The profile of each participant was calculated by converting their emotional exhaustion, cynicism, and professional efficacy scores from the MBI into standardized values (Z-scores) and applying critical thresholds previously defined by Leiter and Maslach [29].

Due to the high number of predictors and low number of observations, several structural factors were removed prior to regression analysis. Pearson correlation was used to determine degree of relatedness of factors; factors with a correlation coefficient > 0.3 were flagged, and two authors decided which factors to exclude based on statistical correlation and contextual significance. In total, 18 factors were excluded. We then aggregated the five profiles into a dichotomized variable (engaged vs. not engaged); not engaged included the ineffective, overextended, disengaged, and burnout profiles. This approach enabled logistic regression to determine the association between various factors and either positive (engaged) or negative (not engaged) profiles.

The narrative responses to the open-ended survey questions and the interview transcripts were analyzed using iterative inductive thematic analysis [30]. Survey responses and interview transcripts were uploaded to ATLAS.ti software. One investigator (KJ) read through all transcripts and generated the codebook inductively (stage 1). To validate the analysis, three authors (NH, NP, TD) separately coded five random interviews using the codebook, and consensus was reached on the final list of codes (stage 2). No conflicting or omitted findings were noted. One author (KJ) reviewed all transcripts with the final codebook (stage 3). Aggregated code reports were generated and reviewed at the completion of the coding process. These were used to develop the major themes and subthemes (stage 4). One author (KJ) continued to analyze and refine all data within each theme and subtheme to ensure that each subtheme was supported by the entire dataset (stage 5). The report was written with input from all members of the research team (stage 6).

## Results

### Quantitative results

Fifty-seven physicians responded to the survey (Table 2). The majority of participants were mid-career family physicians, primarily working in the NWT. Based on the MBI, most participants (*n* = 35) fit into the ‘engaged’ profile, and relatively few into ‘burnout’ or ‘disengaged’ (Table 2).

**Table 2:** Participant characteristics

Characteristic	N
**Age (μ)**	43
**Sex**	
Female	29
Male	28
**Time since training in years (μ)**	13
**Specialty**	
Primary care	38
Medical specialty	8
Surgery	11
**Location**	
Yukon	10
Northwest Territories	44
Nunavut	3
**MBI profile**	
Engaged	35
Overextended	17
Ineffective	3
Burnout	2
Disengaged	0

The ‘engaged’ MBI profile was inversely correlated with use of electronic medical records (β = − 0.7, *p* < .05), inadequate financial remuneration (β = − 1.0, *p* < .05) and cross-cultural issues (β = − 1.1, *p* < .05) (Table 3). Discontinuities in patient care, lack of opportunities for mentorship, and lack of access to research opportunities were not significantly associated with the engaged profile. Similarly, the engaged profile was directly associated with intrinsic love of work (β = 0.9, *p* < .05) and support from coworkers (β = 0.9, *p* < .05) (Table 4). Prior rural experience and support from the local community displayed no significant association with the engaged profile.

**Table 3:** Assessing for contribution to burnout

Factor	β	***p***
Use of electronic health records	−0.71	.037*
Lack of research opportunities	0.28	0.62
Cross-cultural issues	−1.06	0.04*
Discontinuity in care	0.09	0.82
Lack of mentorship opportunities	0.40	0.54
Inadequate financial remuneration	−1.01	0.03*

* indicates significant results (*p* < .05)

**Table 4:** Assessing for protection against burnout

Factor	β	***p***
Prior rural experience	−0.07	0.8
Support from local community	−0.66	0.09
Support from coworker	0.89	0.04*
Love of work	0.87	0.03*

* indicates significant results (*p* < .05)

### Qualitative results

Analysis of the open-ended survey questions and interviews revealed three themes related to burnout: contributing factors, which included health system issues that were perceived as increasing stress among physicians (Table 5); mitigating factors, which participants considered to be protective against burnout (Table 6); and ‘double-edged swords’ — dimensions of northern clinical practice that were valued by participants, but also at times contributed to burnout (Table 7).

**Table 5:** Contributing Factors

Contributing Factors	
Emergent Code	Descriptive Examples
Lack of Influence on Policy and Administration	“I’m realizing policies were all built to exclude physicians – to exclude physicians and physician leadership […] and if you work in an institution that does that, you’re going to burn out all your docs and they’re going to leave.”
	“Physicians have this recurring sense of feeling consumed or used like a product or a resource rather than being treated as a human - which is ironic as our goal is to treat our patients with dignity as human beings, but we don’t feel our work, for structural or interpersonal reasons, gives us the same dignity.”
Systemic Failures in Cultural Safety	“This is a particular problem with locums coming in and not understanding the North. I’ve had to really self-educate on cultural competence because it wasn’t really a thing when I first arrived, and that can definitely contribute to burnout because it contributes to so many communication issues and problems with patients. I think now the government is actually recognizing the need and I actually just attended my first official cultural competence training this week. And that actually felt really good – that my employer actually cared enough to allow me to schedule two days off from my clinical duties, protected time, to actually learn these things formally instead of just learning by trial and error.”
Discontinuity of Care	“Because we’re not adequately resourced to serve our population, we end up having this hamster wheel where we’re racing to meet the access needs of the population rather than supporting continuity of care.”
Upshifting of Tasks	Doctors are carrying a lot of administrative burden – hours and hours of it … It’s very demotivating to do work that’s not only not at the top of your scope, but shouldn’t really be within your work description at all. It’s morale-killing.”
Physician Turnover	“One of the issues is that almost everyone who works here is really working in a sort of expatriate position because they’re working with such a large Indigenous population. They see themselves as coming here for 5 years, making some money and leaving, or working for the federal government, coming here for 10–15 years, getting a pension and then leaving. They don’t have a real personal stake in the system and how it should work.”
	“I think [we need] an effort to keep people here, to keep people coming back, to see what keeps them here, a recognition from administration that there is a higher value to people who keep coming back and continuity rather than just filling spots with a heartbeat.”
Lack of Systemic Supports	“There technically are programs that physicians can access, but few of them are actually tailored to the situation that we find ourselves in in the North. So for example, through the Canadian medical association, there is a financial planning service, but there’s still not any particular service that speaks to the experience of physicians who practice remotely or in the North – that speaks to our unique financial situation, having a very high cost of living and most of us working in a salary based practice.”
	“Through time, what’s happened is they’ve basically said “if you’re not doing well, it’s because you’re personally not resilient enough. You need to work harder at yoga, meditation, eating right,” all that kind of stuff that’s really important. What is now more recognized in terms of what people don’t do well as physicians is it tends to be more systemic issues, so issues of overwork, lack of being able to have a voice, feeling this moral outrage that they’re unable to provide the kind of care they know they need to but being medically responsible if the care is bad; those sorts of things that cause poor physician health. And there’s been a complete lack of understanding up here in regard to those issues.”

**Table 6:** Protective Factors

Protective Factors	
Emergent Code	Descriptive Examples
Relationships	“You know in a work environment where it can be really intense sometimes, if you know everyone personally by name and know their family, they’re much more likely to come in and help each other than I’ve had in a larger place.”
Time on the Land & Outdoor Activities	“It’s such a vast place and it’s really beautiful in many ways so you have a connection with raw nature that you don’t see in many parts of the world so that’s unique. It’s difficult to find areas like this anywhere else in the world.”

**Table 7:** Double-Edged Swords

Double-Edged Swords	
Emergent Code	Descriptive Examples
Scope of Practice	“I’m encouraged to work to my full scope, I can do multiple different things, I don’t just have to come to the office every single day and see people in clinic. I can do procedure, I can push my scope, I can push my boundaries, I can vary my practice.”
	“Always dealing with high acuity, high emergency situations because we don’t see a lot – like big traumas, you know, people who you have to run a code on who are crashing. I was actually talking to a colleague about this recently; one of the hardest aspects of practicing in a rural or remote setting is dealing with babies. Newborn babies who are sick or premature newborn babies – we don’t have pediatricians, we don’t have anybody with specific pediatric training … we put this pressure on ourselves and yet we don’t have the expertise – or we feel like we don’t have the expertise – to adequately manage them. Which is compounded by the fact that in a very remote settings, you’re forced sometimes to care for these patients for a very long time, which would never happen in other settings.”
Blurring Boundaries	“Being able to see the outcomes of your efforts within your community – you’re actually able to see your patients out and about and how they’ve benefitted or seeing the positive steps that they’ve taken for their health.”
	“So doctors have sort of agreed to soft call without any boundaries or limitations on it, so what started out as this very exciting opportunity to do anything and everything has turned into this life-consuming experience. I think what I’ve seen is that there are no boundaries on physician time. And unfortunately there’s this perception that if you pay people well, you can ask them to do anything.”
Time Away	“At any given time, I would say the majority of physicians are near burnt out. The way they schedule us, when you’re in town you work really hard and don’t really have time to have extra socialization with your colleagues or have good balance. And so they take long breaks and leave town -- they go on these really long holidays so we don’t see them then either...So, even though you know that there’s other physicians around you, you don’t necessarily feel that you’re balanced and have time for each other even when you’re in the same building.”
	“That’s probably the only way I survive my job is not being there all the time.”

### Contributing factors

#### Lack of influence on policy and administration

Physicians described the lack of influence they felt they had in the health system due to administrative structures, lack of transparency, and lack of direct involvement in health policymaking. This left many physicians feeling powerless against systemic forces that affect patient outcomes.

#### Systemic failures in cultural safety

Physicians were overwhelmingly critical of the systemic failures to provide culturally safe care and meet the needs of Indigenous patients: “[The health system] feeds into the colonial system of mistrust” leading to “tremendous alienation of Indigenous patients.” Participants remarked they lacked the resources and support to build relationships that align with Indigenous values. Participants cited organizational features such as fifteen-minute appointments; lack of education about Indigenous cultures, history, and experiences; and minimal Indigenous representation in the healthcare workforce as contributors to systemic failures. Furthermore, participants noted that communication via interpreters can help address language barriers but does not allow time to delve into deeper interpretations of patient experiences.

#### Discontinuity of care

Physicians expressed frustration with their inability to provide continuous care to their patients, attributed mainly to the ratio of providers to the population size and distribution. Participants described care teams as understaffed and therefore unable to follow patients longitudinally. Rather than delivering patient-centered care, physicians instead navigate short, episodic interactions.

#### Upshifting of tasks

Providers expressed frustration with the quantity of work assigned to them that falls outside their scope of practice. Tasks including rooming patients, entering labs into the electronic medical record, and cumbersome paperwork processes leave physicians feeling “overstretched” and “drained.”

#### Physician turnover

Physicians attributed some continuity of care challenges to high rates of physician turnover and reliance on locum providers. Participants distinguished between locum providers who return to a single site for many years and become a consistent part of the clinical team, as compared to those who only come for a short period, require significant orientation and training investments, and are seen as providing lower quality, less efficient care. Participants explained that short-term locums often lack relationships with the community and the ability to follow-up with patients after they leave, which adds to the burden on permanent providers.

#### Lack of systemic supports

Participants noted services such as physician health programs, continuing education, and financial planning to be lacking in the North. Though some southern programs offer remote support for northern physicians, they are not tailored to the North’s unique context.

### Mitigating factors

#### Relationships

The most commonly reported protective factor was the relationships physicians have with their colleagues and communities. Participants reported that they often felt buoyed by colleagues and said being able to count on one another mitigates feelings of isolation. “There’s a strong sense of collegiality...of ‘we’re in this together.’”

#### Time on the Land & Outdoor Activities

Participants explained that a valuable component of their experience in the North is the ability to spend time on the land. Time spent in nature allows physicians to disconnect from their work life. This environment also provides a setting for physicians to build deep and meaningful relationships with their community, and to witness Indigenous cultures and gain context for what health means to the people they serve.

### Double-edged swords

#### Scope of practice

Northern physicians maintain a broad scope of practice which many described as a motivating factor that keeps their work engaging. A major determinant of physicians’ perspectives on their scope of practice was the amount of support they received from both family physicians and specialists; if participants felt safe with a procedure and could call for help when necessary, they reported feeling confident with a broad scope. Conversely, if they felt thrust into situations beyond their comfort level with inadequate support, the same broad scope became stressful.

#### Blurring boundaries

The territories provide a unique practice environment in which healthcare providers become integral members of the communities they serve. Because most of these communities are small, physicians often see their patients outside the clinic. This can be immensely fulfilling for providers, making them feel connected to their patients, and allowing them to “see the outcomes of [their] efforts within their community.” However, those encounters and relationships become taxing when pushed too far – receiving questions via social media, diagnosing a colleague, being ignored by a patient who suffered a bad outcome. As the boundaries between work and personal life blur, physicians begin to feel “always on.”

#### Time away

Though vacation time emerged as a protective factor, providers argued it is used as a post hoc solution to issues of overscheduling. By interfering with relationship building and interrupting continuity of care, time away represents a suboptimal and superficial remedy to deeper problems.

## Discussion

This study sought to characterize physician burnout in the three northern territories in Canada. We identified structural factors associated with the risk of burnout, including extensive use of electronic medical records, inadequate financial remuneration, and systemic cross-cultural issues. Overall, the findings of the qualitative analysis supported the quantitative results, and further elucidated the drivers of burnout in the context of northern health care. Participants explained that their limited influence on regional health care policies and systemic failures that disallow the provision of a culturally safe clinical setting have a negative impact on physician wellbeing.

Sixty-one percent of respondents were grouped into the ‘engaged’ MBI profile, and 29% fit in the ‘overextended’ profile. The ‘overextended’ profile presents in a physician who is dedicated to and derives a strong sense of professional efficacy from her work but is extremely tired [29]. The qualitative analysis substantiated this finding: physicians described heavy workloads and understaffing as significant issues affecting burnout. Physicians advocated for administrative focus to shift away from recruitment to instead focus on the retention of dedicated, competent providers.

Our findings demonstrate that burnout among northern physicians is multifactorial and has roots in context-specific issues in northern health systems. The limited global literature on burnout amongst physicians in other rural settings reveals similar findings. A study of rural providers in Iran reported that physicians felt “unimportant in the system” and lacked autonomy. In turn, these feelings threatened relationships with the community and contributed to burnout [12]. In rural Japan, dissatisfaction with income was related to increased burnout and intention to resign, while support from co-workers and a high degree of professional autonomy were related to decreased burnout [31]. In Australia, a “fly-in fly-out” healthcare model, akin to the locum system in Canada, contributed to burnout [32]. This was also found to be the case in our study. Furthermore, factors previously identified as important for retention of health professionals in Nunavut, including aligning service models with population needs and incentivizing long-term commitment to the region [33], were similarly identified as burnout mitigators in this study.

While cultural insensitivity has been shown to negatively affect patient experiences [34], our results suggest it is also an important driver of physician burnout. Health systems in the North are designed and governed by knowledge and values that rarely reflect those of the Indigenous communities they are meant to serve [35, 36]. Several studies have reported that Indigenous peoples in the territories experience interpersonal and systemic racism in the health system [37]. Physicians in northern Canada routinely bear witness to their patients’ grief and frustration toward institutional racism within the healthcare system, though their profession, as a part of this system, inherently maintains a level of complicity in propagating such inequities [38]. Similar findings in Australia reported that addiction medicine workers bore an enormous emotional burden as their Aboriginal patients shared experiences with racism and stigma [39]. The system’s refusal to respond to Indigenous patients’ cultural needs directly thwarts individual physician efforts at providing culturally responsive care. Furthermore, deliberate exclusion of Indigenous and physician voices from administrative decision-making forestalls progress and contributes to physicians feeling complicit in a flawed system they are unable to change.

Future research should explore cross-cultural communication and structural racism as potential drivers of burnout and workforce attrition. Much of global health practice occurs across cultural differences, and recent recognition of burnout as a smoldering global health crisis should prompt greater investigation into the role of cultural barriers in such challenges [14, 40].

### Limitations

This study was impacted by several limitations. The small sample size and low response rate meant that the results had low statistical power. One factor that likely impacted the response rate was the territory-wide ransomware attack in Nunavut that occurred at the time the survey was distributed. Higher rates of participation, especially from physicians in Nunavut and Yukon would have increased the statistical strength of the study and may have influenced the results. The use of mixed methodology enabled us to partially overcome this limitation by facilitating greater accuracy and completeness of our findings.

Another major limitation was the possible selection bias. Physicians who were most likely to be burned out may have been the most likely to have left the North or not be licensed, and therefore would no longer be eligible to participate. The sampling method used to identify participants for qualitative interviews depended upon their completion of the quantitative survey. Furthermore, individuals opting to participate in interviews or respond to open-ended questions in the survey may have been more likely to harbor strong opinions about burnout.

While incorporating qualitative interviews was immensely valuable in contextualizing quantitative findings, it was also inherently limited. Though fourteen interviews achieved saturation of ideas, there are certainly perspectives within Northern Canada that were not captured. Additionally, the nuanced context and findings captured in a qualitative approach may be less easily translated to other contexts. While these limitations were partially overcome by applying a mixed methods approach, physician burnout in rural and remote settings requires further exploration, particularly as we look ahead towards identifying solutions.

## Conclusion

Lack of physician influence on administrative policy and decision-making, and lack of Indigenous cultural responsiveness by the health system emerged as actionable drivers of physician burnout. Heavy workloads and understaffing further contribute to the ‘overextended’ burnout profiles identified. The results of this study may have implications for other rural or resource-constrained global clinical settings.

## Supplementary Information

**Additional file 1.**

## Data Availability

The datasets generated and analyzed during the current study are available from the corresponding on reasonable request.
