# Supplementary material for: Exploring the impact of rural health system factors on physician burnout: a mixed-methods study in Northern Canada
Source: BMC Health Serv Res. 2021 Aug 25;21:869. doi: 10.1186/s12913-021-06899-y (PMC8390267; doi:10.1186/s12913-021-06899-y)
Supplement: Supplementary file 1 — Additional file 1. [file 12913_2021_6899_MOESM1_ESM.docx]

**Title:** Exploring the impact of rural health system factors on physician burnout: A mixed methods study in northern Canada

**Names and affiliations of authors:** Nathaniel Hansen, BS^1^; Kennedy Jensen, BA^2^; Ian MacNiven, MD^3^; Nathaniel Pollock, PhD^4^; Thomsen D’Hont MD^5^, Susan Chatwood, BScN, MSc, PhD^6^

**Affiliations:**

1. Tufts University School of Medicine, Boston, Massachusetts, USA.
2. Geisel School of Medicine at Dartmouth, Hanover, New Hampshire, USA
3. Northwest Territories Health and Social Services Authority, Yellowknife, Northwest Territories, Canada
4. School of Public Health, University of Alberta, Edmonton, Alberta, Canada
5. Department of Family Medicine, Faculty of Medicine, University of Alberta, Yellowknife, Northwest Territories, Canada
6. School of Public Health, University of Alberta, Yellowknife, Northwest Territories, Canada

**Corresponding author contact information:**

Nathaniel Hansen, BS

Tufts University School of Medicine

Boston, Massachusetts, USA

(207)-939-6576 | nathaniel.hansen@tufts.edu

**Appendix A: Open Ended Survey Questions**

1. Is there anything related to the cultural responsiveness of the health system that impacts your satisfaction as a provider? If so, please expand.
2. Are there any factors that might contribute to or be protective against burnout that are not listed above? If so, please expand.

**Appendix B: Semi-Structured Interview Guide and Qualitative Methodology Details**

1. To begin, tell me a little bit about your medical career and what brought you to the North? What kept you here?
2. Could you speak to some of the aspects of practicing medicine in the North that you find fulfilling or keep you motivated?
3. On the flip side, could you speak to some of the aspects of practicing medicine in the North that you find particularly draining?
4. Are you familiar with the idea of physician burnout? What do you think physician burnout looks like for doctors where you practice?
5. What is your perspective on cultural sensitivity/cultural competence? How has that impacted your experience practicing medicine in the North?
6. If you had both the ability and resources to make some sweeping changes in the system, what would your priorities be?

*All interviews were conducted by KJ, a female medical student with training in qualitative research methods. The author had no prior relationship with study participants prior to conducting interviews. Her lack of experience practicing medicine in the North prevented significant bias from influencing conversations with participants. All participants were made aware of the author’s status as a medical student prior to the interview. No repeat interviews were conducted. All quotations used in this manuscript were first returned to participants for comment and correction.*
